# Description of the male of *Leptotyphlus kovaci* Šustek, 2000, the only Central European species of the Mediterranean genus *Leptotyphlus* Fauvel, 1874 (Coleoptera, Staphylinidae, Leptotyphlinae)

**DOI:** 10.3897/zookeys.509.10059

**Published:** 2015-06-25

**Authors:** György Makranczy

**Affiliations:** 1 Department of Zoology, Hungarian Natural History Museum, H-1088 Budapest, Baross utca 13, Hungary

**Keywords:** *Leptotyphlus kovaci*, male characters, Silicka planina, Jelšavský kras

## Abstract

The previously unknown male of *Leptotyphlus kovaci* Šustek, 2000 is described and illustrated. The relationship of the species is discussed. The species is also reported from Gemerskoteplická jaskyňa near Jelšavská Teplica (Slovakia).

## Introduction

The genus *Leptotyphlus* Fauvel, 1874 with more than 200 named species (Herman 2001 and subsequent descriptions) is the most speciose genus of the subfamily Leptotyphlinae (worldwide distribution, 43 genera, 515 species). Members of the subfamily are adapted to subterranean life and soil-dwelling; they are frequently found near caves. The known distribution of *Leptotyphlus* is from the Pyrenees and Southern France to Italy, with a few species in Spain and Tunisia. With its predominantly Western and Central Mediterranean distribution, it was a great surprise when a new species of this genus was described from Ardovská jaskyňa at Ardovo (Pelsőcardó), Slovakia, not far from the Hungarian border (Šustek 2000). While there are no real gaps in the known distribution of this genus (only about 150 km), *Leptotyphlus kovaci* Šustek, 2000 was found about 630 km away from the nearest other member of this genus (*Leptotyphlus foroiuliensis* Pace, 1976). The description was based on two females (in microscopic preparation). The specimens came from soil at a forested cave entrance of Ardovská jaskyňa near the village Ardovo, a few kilometers SE of Plešivec. Attempts to gather more specimens remained unsuccessful until in 2009 the present writer collected 4 specimens, including one male, at the type locality. In 2010 the species was also found at Gemerskoteplická jaskyňa near Jelšavská Teplica. In contrast to the type locality, this is a watery cave, with a stream exiting and providing humid microclimate, not allowing the soil to dry out. The description of the male is very important, since this species is the only Central European member of this very speciose genus, and also because only based on characters of the aedeagus can its phylogenetic affiliations be assessed.

## Material and methods

In the framework of the Gemer-ATBI+M program (EDIT WP7), the author had an opportunity to collect at the type locality on two occasions. The cave is situated at the edge of Silicka planina (Szilicei-fennsík). The new record is from Jelšavský kras, an adjacent large karstic area. For collecting, the soil-washing method was used: soil together with roots and stones is immersed in water, repeatedly stirred, and the substance gathering on the water surface is collected with a fine tea-filtering net. The organic matter was gathered in a fabric sack which was then drained from excess water and the material was eventually placed in Berlese funnels for further drying and was heated with small (20W) lamps from above. Sampling was done at 6-7 different spots per site to increase the chance of capture. The specimens are dry mounted. Genitalia drawings were made by embedding into Euparal mounting medium on small plastic slides pinned with the specimens. Drawing was done with a Jenalab (Carl Zeiss, Jena) compound microscope and drawing tube (camera lucida), SEM imaging of uncoated specimens with a Hitachi S-2600 N scanning electron microscope. The terminology of the description follows Orousset (1987, 2013).

The following codens indicate collections in which the listed specimens are deposited: The Natural History Museum (formerly British Museum of Natural History), London, United Kingdom (BMNH), Hungarian Natural History Museum, Budapest, Hungary (HNHM). Numbers in brackets “{ }” stand for collecting events.

## Taxonomy

### Leptotyphlus (Leptotyphlus) kovaci

Taxon classificationAnimaliaColeopteraStaphylinidae

Šustek, 2000

Figs 1–6 , 7–10

Leptotyphlus (Leptotyphlus) kovaci Šustek, 2000: 151.

#### Material examined.

SLOVAKIA, Silická planina, 1 km SSE Ardovo, Ardovská jaskyňa, dark oak-maple forest at cave entrance, 310 m, top 15 cm of soil (roots, stones, humus), soil-washing {016}, 48°31’18”N, 20°25’16”E, 30.V.2009, leg. Gy. Makranczy (1 male, 2 females, HNHM, 1 female, BMNH); SLOVAKIA, Jelšavský kras, Gemerskoteplická jaskyňa, 1.5 km E Jelšavská Teplica, forest at cave entrance, 230 m, soil-washing 1-4 m from outcoming stream, 20 cm deep {115}, 48°36’18”N, 20°17’42”E, 25.IV.2010, leg. Gy. Makranczy (1 male, HNHM).

#### Partial redescription

**Figures 1–6.**
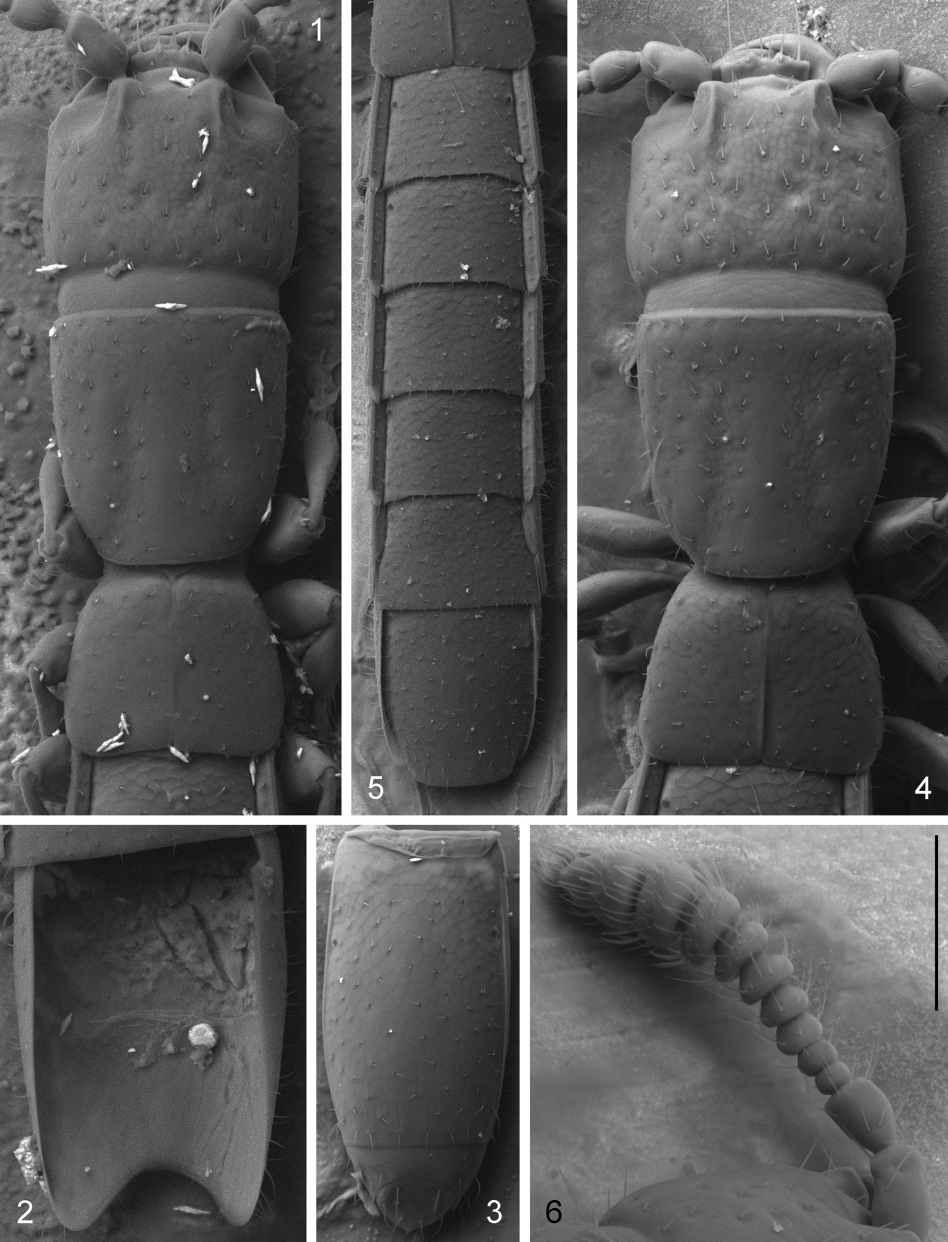
*Leptotyphlus kovaci* Šustek, 2000 male from Ardovská jaskyňa (**1–3**) and male from Gemerskoteplická jaskyňa (**4–6**). **1, 4** forebody **2** sternite VIII, dorsal view **3** abdominal segments IX–X, ventral view **5** abdomen, dorsal view **6** antenna. Scale bar: 0.06 mm (**6**), 0.08 mm (**2**), 0.1 mm (**1, 4**), 0.11 mm (**3**), 0.15 mm (**5**).

**(male).** Measurements in mm (separately for male from Ardovská jaskyňa / male from Gemerskoteplická jaskyňa): head width = 0.14 / 0.155; maximum width of pronotum = 0.135 / 0.15; maximum width of elytra = 0.12 / 0.14; maximum width of abdomen = 0.14 / 0.15; head length from front margin of clypeus to the beginning of neck = 0.14 / 0.14; length of pronotum in the middle-line = 0.14 / 0.16; length of elytra from hind apex of scutellum = 0.09 / 0.10; length of abdomen = 0.71 / 0.75 (all measured from dorsal view). Forebody as in Fig. 1 (Ardovská jaskyňa) and Fig. 4 (Gemerskoteplická jaskyňa). Head subrectangular, length about 4/5 of width, parallel-sided, neck separated by transversal groove. Pronotum very slightly narrower than head, both anterior and posterior pronotal margins truncate, sides narrowing behind, gently arched anteriorly, more strongly on posterior portion; anterior angles narrowly rounded, posterior angles more broadly. Elytra together less broad than pronotum, trapezoid, shoulders not developed, posterior margin almost truncate, very slightly concave at suture. Abdomen (Gemerskoteplická jaskyňa) as in Fig. 5, very elongate and parallel-sided; segment VII with much shortened inner laterosclerites. Antenna (Gemerskoteplická jaskyňa) as in Fig. 6, antennomeres 3–11 strongly transverse, articles 9–11 with modified setae. Dissected genital segments of male from Ardovská jaskyňa: sternite VIII (dorsal view) as in Fig. 2, segments IX–X (ventral view) as in Fig. 3.

**Figures 7–10.**
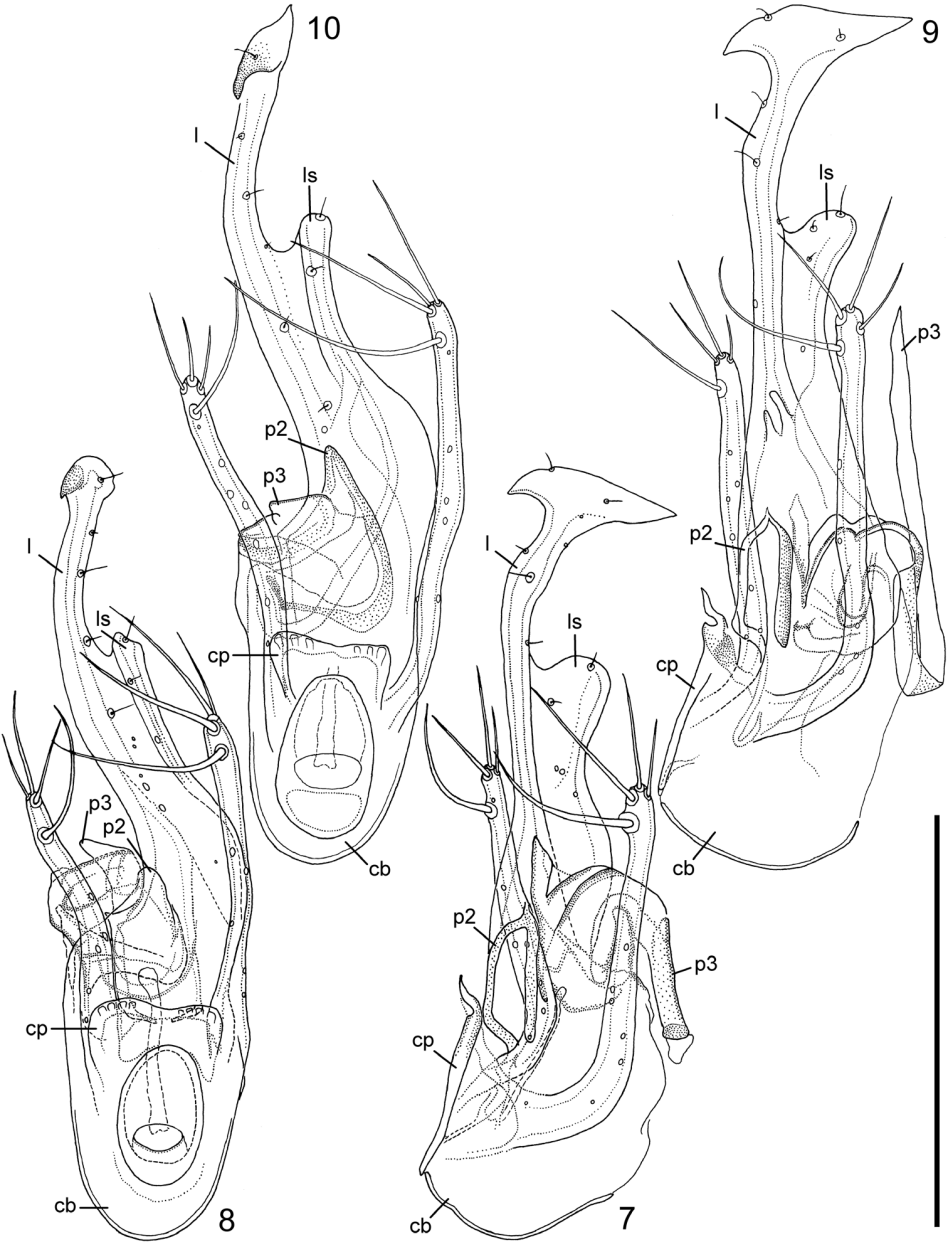
*Leptotyphlus kovaci* Šustek, 2000 male from Ardovská jaskyňa (**7–8**) and male from Gemerskoteplická jaskyňa (**9–10**). **7, 9** lateral view **8, 10** “parameral” view. Scale bar: 0.1 mm.

Aedeagus (Figs 7–10). Greatly asymmetrical. Sternal lamina (l) strongly developed, elongate, with a broadened, hammer-like apex. Sternal lobe (ls) well-developed, in lateral view on the side of sternal lamina opposite to basal orifice, exceeding half length of sternal lamina; in parameral view sternal lobe situated at right side of sternal lamina. Both left and right parameres well-developed, each with 4 strong setae on apex; apices approaching but not reaching half of length of sternal lamina from basal capsule. Copulatory pieces: p1 not developed (in most other related species a large, broad blade-like structure, often approaching apex of sternal lamina), p2 present but weakly developed, without any peculiar formation, p3 (often helicoid in related species) present but inconspicuous (a little sticking out in the Gemerskoteplická jaskyňa specimen, but can be explained as variability). Proximal callus (cp) on basal capsule (cb) very strongly developed, apically with a deflexed edge.

Sexual dimorphism: none besides the usual differences in terminalia. The much wider genital segments mentioned in the original description are an artefact resulting from squeezing the specimens by mounting them between glass surfaces.

#### Remarks.

Based on the structures of the male genitalia, the species belongs to the subgenus *Leptotyphlus* as correctly stated in the original description and to the Leptotyphlus (Leptotyphlus) tyrrhenius species group (sensu Pace (1996)). Similar species are Leptotyphlus (Leptotyphlus) uccellinensis Pace, 1978 and Leptotyphlus (Leptotyphlus) aithaliensis Orousset, 1983, but in Leptotyphlus (Leptotyphlus) kovaci the sternal lobe is on the opposite side of the sternal lamina, compared to most other known species, and the p1 lobe of copulatory pieces is not developed. The sole male specimen from Gemerskoteplická jaskyňa is slightly larger, with somewhat broader head. However, the comparison of the aedeagi reveals only minor differences that may be due to infraspecific variation or distortions. There is no evidence suggesting that the two male specimens may represent different species. On the other hand, the aedeagus of Leptotyphlus (Leptotyphlus) kovaci differs quite remarkably from the most similar congeners in Tuscany and Corsica. The knowledge of the male genitalia may gain greater significance when more species are discovered, especially from more southern mountain ranges of the Carpathians or from the northern Balkans.

## Supplementary Material

XML Treatment for Leptotyphlus (Leptotyphlus) kovaci
